# Atherosclerosis: A Comprehensive Review of Molecular Factors and Mechanisms

**DOI:** 10.3390/ijms26031364

**Published:** 2025-02-06

**Authors:** Vasiliki Tasouli-Drakou, Ian Ogurek, Taha Shaikh, Marc Ringor, Michael V. DiCaro, KaChon Lei

**Affiliations:** 1 Department of Internal Medicine, Kirk Kerkorian School of Medicine at the University of Nevada, Las Vegas, NV 89106, USA; ian.ogurek@unlv.edu (I.O.); taha.shaikh@unlv.edu (T.S.); marc.ringor@unlv.edu (M.R.); michael.dicaro@unlv.edu (M.V.D.); 2 Department of Cardiovascular Medicine, University of Nevada, Las Vegas, NV 89106, USA; kachon.lei@unlv.edu

**Keywords:** atherosclerosis, cardiovascular health, inflammation

## Abstract

Atherosclerosis, a condition characterized by the accumulation of lipids and a culprit behind cardiovascular events, has long been studied. However, in recent years, there has been an increase in interest in its initiation, with researchers shifting focus from traditional pathways involving the vascular infiltration of oxidized lipids and towards the novel presence of chronic inflammatory pathways. The accumulation of pro-inflammatory cytokines, in combination with the activation of transcription factors, creates a positive feedback loop that drives the creation and progression of atherosclerosis. From the upregulation of the nod-like receptor protein 3 (NLRP3) inflammasome and the Notch and Wnt pathways to the increased expression of VEGF-A and the downregulation of connexins Cx32, Cx37, and Cx40, these processes contribute further to endothelial dysfunction and plaque formation. Herein, we aim to provide insight into the molecular pathways and mechanisms implicated in the initiation and progression of atherosclerotic plaques, and to review the risk factors associated with their development.

## 1. Introduction

Atherosclerosis is well-established as the main driving force behind cardiovascular disease (CVD) and related vascular diseases, such as coronary artery disease (CAD), peripheral arterial disease (PAD), cerebrovascular accidents (CVAs), aortic atherosclerotic disease, and more. Collectively, these diseases are termed atherosclerotic cardiovascular diseases (ASCVDs). Overall, ASCVD is the leading cause of death worldwide [1]. Although the mechanism of atherosclerosis has been studied over the years, new risk factors for its development and progression have come to light.

Recently, there has been a paradigm shift surrounding the development and progression of atherosclerosis, which has challenged conventional thinking. Atherosclerosis is now regarded as a chronic inflammatory condition in which macrophages infiltrate atherosclerotic sites, accumulate lipids, and form into foam cells while secreting inflammatory mediators [2]. Although certain demographics of patients are more predisposed to the development of cardiovascular disease than others, the complex inter-relationship between atherosclerosis and molecular factors should not be ignored [3]. Similarly, the complex nature of atherosclerosis has called for the development of emerging therapies, such as the use of small interfering RNAs (siRNAs) as a novel therapeutic strategy to prevent the assembly of lipoproteins and reduce the effects of cardiovascular risk factors and diseases [4,5].

This review aims to provide a comprehensive summary and analysis of the best available evidence surrounding the cellular and molecular mechanisms implicated in the development and progression of atherosclerosis. We will explore key cellular processes involved in the pathogenesis of atherosclerosis, particularly those related to endothelial dysfunction, lipid oxidation, and immune cell infiltration. Finally, we explore state-of-the-art recommendations and guidelines in the context of the novel view of ASCVD, most specifically related to CAD.

## 2. Pathophysiology

### 2.1. Anatomy

Blood vessels are composed of three layers. The innermost layer, the tunica intima, is composed of endothelial cells (ECs) that line the vessel lumen and serve as a selectively permeable barrier, as well as smooth muscle cells (SMCs) and pericyte-like stellate cells [6,7]. Superficial to the tunica intima is the tunica media, composed of SMCs and extracellular matrix (ECM) components like elastin and collagen [6]. The tunica media helps regulate the vascular tone and provides structural integrity to the vasculature. The tunica media is surrounded by the tunica adventitia, which is composed of loose ECM, fibroblasts, nerves, and small arteries known as the vasa vasorum and which plays a role in the growth and repair of arteries [6]. Interestingly, the distance or thickness between the intimal–luminal and the medial–adventitial surface of the carotid artery, known as the carotid intima-media thickness (IMT), commonly serves as a marker of atherosclerosis, with thicknesses larger than 1.5 mm generally indicating the formation of an atherosclerotic plaque [8].

### 2.2. Important Signaling Pathways

To comprehend the pathophysiology of atherosclerosis, it is vital to understand the mechanisms leading to its development. Atherosclerosis is characterized by the accumulation of lipids and fibrous elements in large arteries [9]. Very low-density lipoproteins (VLDLs) synthesized by hepatocytes, move into the blood through pores in ECs of the liver sinusoids and arrive at the capillaries, where they come into contact with chylomicrons to deliver cholesterol and fatty acids to the apical membrane of ECs there [10]. After delivering cholesterol, VLDLs are transformed into low-density lipoproteins (LDLs), which are transported and penetrate through continuous endothelium into the tunica intima. Usually, continuous endothelium lining the arteries is impermeable to LDLs, but holes within it can be created through the presence of turbulence or mechanical stress, the latter of which is discussed in greater detail below.

One driving mechanism behind the reason why LDLs penetrate the intima is the process of de-sialylation discovered by Orekhov et al. in 1989 [11]. They found that when they de-sialylated LDLs (sialic acid is a component of native LDLs) by incubating them with sialidase and added them to human aortic intimal cells of SMC origin, they saw not only an increase in cholesteryl esters, triglycerides and free cholesterol, but also that cultured cells degraded de-sialylated LDLs more readily than native LDLs. This finding led them to conclude that alterations in the composition of LDLs rather than elevation in plasma LDL levels were more likely to predispose people to atherogenicity. This phenomenon may explain why individuals with CAD have significantly less sialic acid in their LDL particles compared to individuals without CAD [12].

When LDLs in the form of low-density lipoproteins (LDLs) become internalized in the tunica intima, they activate ECs, a process that is mediated by scavenger receptor B1 (SR-B1) and activin A receptor-like type 1 (ALK1) receptor [13]. The infiltration of LDLs into the tunica intima and the ECM further initiates the recruitment of monocytes and their transformation into macrophages (the M1 phenotype) in the subendothelial space [14]. In the intima, LDLs become oxidized by reactive oxygen species (transforming into oxLDLs), and induce uptake by macrophages leading to the formation of foam cells whose creation is further discussed below [15]. The binding of sialic acid in LDLs to the receptor sialic acid-binding immunoglobulin-like lectin (Siglec) found in many white blood cells stimulates the progression of atherosclerosis [16]. One cell of interest is B-lymphocytes of the B-1 subtype, the binding of which to sialic acid further inhibits their atheroprotective effects. B-1 lymphocytes produce oxLDL-specific IgM to inhibit scavenger receptor-mediated uptake of oxLDL by macrophages. Several studies have also indicated that toll-like receptors (TLRs) play a role in the recruitment of macrophages to the arterial wall during the development of atherosclerosis, with adaptor protein MyD88 mediating the signaling of all TLRs except TLR3 [17]. Special significance has been given to the TLR4 and TLR2 receptors, with trials showing reduced lesion size in mice deficient of TLR4 and TLR2 [18].

MyD88 is found in immune cells and macrophages, and can alter EC function when it comes to cardiovascular cell types [19]. It contains a unique domain known as toll-IL1R (TIR), which allows it to associate with TLRs and the IL-1R family. It can dimerize through its death domain (DD) when it binds to either receptor and recruits the IL-1R associated kinases (IRAKs) IRAK4 and IRAK1/2, forming a complex called the Myddosome [19]. Upon formation of the Myddosome, the kinase-like domains of IRAK1 and IRAK2 are in close proximity, inducing their phosphorylation, subsequent activation, separation from the Myddosome, and interaction with the tumor necrosis factor receptor-associated factor 6 (TRAF6) [17]. This new IRAK-TRAF6 complex binds to another complex comprised of transforming growth factor activated kinase (TAK1), TAK1-binding protein 1 (TAB1), and TAK1-binding protein 2/3 (TAB2/3) [20]. The activated TAK1 is capable of activating the inhibitor of nuclear factor-kappa B (IκB) kinase complex (IKK complex), which phosphorylates the IκB proteins and degrades them. This process leads to the release and translocation of nuclear factor-kappa B (NF-κB) to the nucleus, thereby inducing the transcription of genes that play a role in the production of pro-inflammatory cytokines. Tumor necrosis factor (TNF-α), IL-6 and IL-8, matrix metalloproteinases (MMPs)-1, 3, and 9, and tissue factor (TF) are among the pro-inflammatory molecules controlled by NF-κB [21]. Important receptors of the IL-1R family involved in atherosclerosis include IL-1R and IL-18R [22]. The secretion of pro-inflammatory cytokines triggers the release of other pro-inflammatory molecules, such as T- and B-lymphocytes, dendritic cells (DCs), and vascular smooth muscle cells (VSMCs) [23]. Cytokine-induced intercellular adhesion and vascular cell adhesion molecules ICAM-1 and VCAM-1 are potent inducers of the recruitment and adhesion of leukocytes in vascular endothelial cells [24].

**Figure 1:**
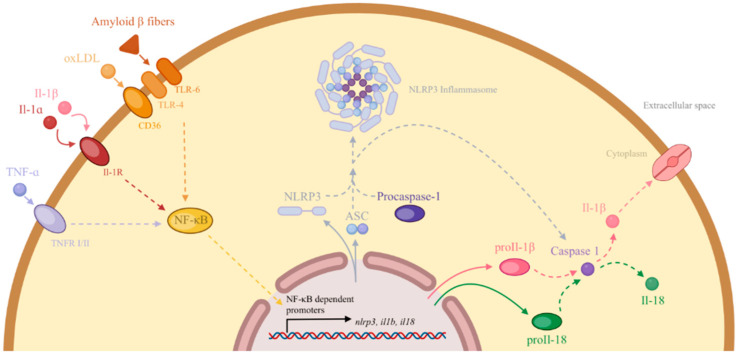
Figure showing the creation of the NLRP3 inflammasome and pro-inflammatory cytokines IL-1β and IL-18. The attachment of oxLDL on the CD36–TLR4–TLR6 signaling complex activates NF-κB, which translocates to the nucleus and binds to NF-κB-dependent promoters for the activation of target genes. The produced NLRP3 binds with an apoptosis-associated speck-like protein (ASC) and procaspase-1 to create an NLRP3 inflammasome. The NLRP3 inflammasome cleaves pro-caspase-1 into its active form caspase-1, which in turn cleaves pro-IL-1β and pro-IL-18 to their active isomers IL-1β and IL-18, respectively. It is important to note that the created IL-1β can bind to IL-1R on the extracellular surface of macrophages to further induce the formation of more IL-1β and IL-18, thus creating a pro-inflammatory environment [27].

As previously mentioned, once oxLDLs are formed, they are scavenged by activated macrophages, the latter of which become lipid-laden foam cells. Macrophages can sense oxLDLs through a variety of receptors, such as CD36 and SR-A1 (their transcription is regulated by NF-κB and downgraded by anti-atherosclerotic messengers like polyphenols and curcumin), macrophage receptor with collagenous structure (MARCO), and lectin-like oxLDL receptor-1 (LOX-1), all of whose expression is upregulated in atherosclerosis, leading to increased oxLDL uptake [25]. The accumulation of lipid-rich macrophages and dendritic cells activates a multiprotein complex called the nod-like receptor protein 3 (NLRP3) inflammasome (whose activation occurs when oxLDLs bind to the CD36–TLR4–TLR6 signaling complex on the macrophage surface). These cellular and molecular processes further induce the secretion of pro-inflammatory cytokines IL-1β and IL-18 as demonstrated in Figure 1 [26].

Acyl coenzyme A cholesterol acyltransferase-1 (ACAT1), found in macrophages, is crucial for the formation of cholesterol esters from free cholesterol and for the transformation of macrophages into foam cells. The enzyme neutral cholesterol ester hydrolase (NCEH) is known to maintain equilibrium between the presence of free cholesterol and cholesterol esters by hydrolyzing cholesterol esters to free cholesterol for efflux out of cells [28]. Its expression, however, is downregulated in atherosclerosis. Like NCEH, ATP-binding cassette proteins A1 (ABCA1) and G1 (ABCG1) have also been shown to play a role in maintaining cholesterol homeostasis by eliminating excess cholesterol from cells like macrophages and assisting in the formation of nascent high-density lipoprotein (HDL) [29].

Over time, progressively accumulating foam cells evolve into fatty streaks, whose presence shapes the early atherosclerotic lesion. Fatty streaks mature into plaques that become covered by a fibrous cap composed of VSMCs and ECM molecules such as collagen [30]. The atherosclerotic plaque slowly becomes primarily composed of VSMCs, macrophages, and T-lymphocytes surrounding a lipid-rich necrotic core. Macrophages and T-lymphocytes have a CD40 receptor that, upon interaction with a CD40 ligand (CD40L), induces the release of cytokines IL-1 and interferon-γ (IFN-γ) from macrophages. CD40 ligands are found in cells such as dendritic, endothelial and T-cells and are known to play a role in T-cell differentiation and T-cell-mediated immunity [24]. Studies have shown that atherosclerotic lesions can trigger the loss of VSMCs, ECs, and macrophages through apoptosis (mediated by IFN-γ) [31]. Their death can contribute to atherosclerotic plaque rupture, the root cause of type 1 myocardial infarctions.

Gradually, atherosclerotic plaques become calcified. Calcification can be triggered by various factors, especially in different populations. For example, in type 2 diabetic patients, hyperglycemia and hyperlipidemia have been known to precipitate inflammation and arterial calcification [32]. The calcification process involves the differentiation of VSMCs into osteoblast-like cells via expression of runt-related transcription factor 2 (RUNX2) [33]. RUNX2 upregulates the expression of the ligand RANKL for activating the RANK (receptor activator of NF-κB) receptor. Panizo et al. suggested that the interaction of RANKL with RANK stimulates a pathway that increases expression of bone morphogenetic protein 4 (BMP4), which is involved in the osteogenic transition of VSMCs, leading to vascular calcification [34]. As a result, the calcification of atherosclerotic plaques shares many similarities with osteogenic differentiation, as well as skeletal mineralization. The enzyme tissue-nonspecific alkaline phosphatase (TNAP) is also found to play a role in promoting vascular calcification by decreasing the levels of plasma pyrophosphate, which is an inhibitor of vascular calcification [35].

Special emphasis on the development of atherosclerotic plaques should be given to the location of formation, with plaques more commonly occurring in branched or curved arteries [36]. Anatomically, these sites are predisposed to low or oscillatory endothelial shear stress, thereby disrupting the physiologic laminar blood flow [37]. Moreover, it is in these areas that atherosclerotic plaques become most vulnerable. As previously mentioned, the additional loss of VSMCs and ECs from the fibrous cap via apoptosis makes the plaque more prone to rupture, with the highest risk of rupture occurring in areas where the fibrous cap is the thinnest [31]. In fact, plaque instability is characterized by fibrous cap thickness of less than 65 μm (μm), the presence of a large lipid necrotic core (greater than 40% of total lesion volume), and infiltration of lymphocytes and macrophages [38]. Additionally, Pedrigi et al. suggest that the presence of high wall stress and exacerbation of inflammation through the production of pro-inflammatory molecules also accelerates plaque rupture [39].

Disrupted plaques trigger the activation of platelets for thrombus formation. The Von Willebrand factor forms a bridge between exposed collagen and platelet glycoprotein (GP) Ib-IX-V receptor complexes on the platelet membrane to initiate platelet deposition [40]. Platelet binding is additionally established through the GPIa/IIa and GPVI platelet receptors, which help induce platelet activation upon binding to exposed collagen [41]. Activated platelets change shape, which subsequently triggers the release of adenosine diphosphate (ADP), 5-hydroxytryptamine (5-HT), and thromboxane A2 to support additional platelet recruitment and vasoconstriction [40]. Released ADP stimulates platelet P2Y12 receptors to induce changes in platelet morphology while developing a thrombus, initiating the interaction of platelet receptor GPVI with released collagen. This collagen-GPVI interaction further upregulates the expression of the GPIa/IIa platelet receptor to facilitate the firm adhesion of platelets to the damaged area and their activation [42]. The feedback loop becomes more prominent when taking into consideration that platelets secrete MMP9 and cathepsin G, which break down the ECM and trigger the rupture of the fibrous cap [43].

**Figure 2:**
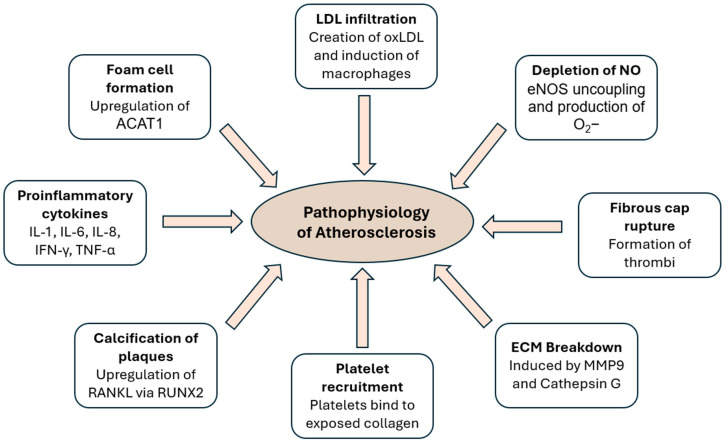
Figure summarizing the pathophysiology of atherosclerosis. From the infiltration of LDLs to the creation of a calcified fibrous cap and its rupture, multiple factors contribute to the initiation and progression of atherosclerosis.

The rupture of atherosclerotic plaques and thrombus formation can manifest as acute ischemic events. This occurs as growing thrombi become occlusive, compromising blood flow to the myocardium [44]. Acute coronary syndrome (ACS) may become exacerbated by vasoconstriction, whether induced by platelet-derived factors, thrombin, or systemic catecholamine release precipitated by the stress event [45]. Thrombi might also remain small, going unnoticed. The pathophysiology of atherosclerosis is briefly summarized in Figure 2 below.

### 2.3. The Role of Nitric Oxide

Nitric oxide (NO) produced by endothelial NO synthase (eNOS) promotes VSMC relaxation and inhibits platelet aggregation [46]. This is achieved when NO activates the enzyme soluble guanylate cyclase (sGC) in VSMCs to catalyze the conversion of guanosine triphosphate into cyclic guanosine monophosphate (cGMP) and activated protein kinase G (PKG). Separate cascades of reactions become triggered. NO exerts antioxidant effects by upregulating the expression of heme-oxygenase-1, ferritin, and superoxide dismutase, the last of which catalyzes the conversion of free radicals in the form of the superoxide anion (O_2_−) into hydrogen peroxide (H_2_O_2_) [47]. NO is known to have a very short half-life, and in conditions like atherosclerosis, where there is a greater amount of oxidative stress, NO becomes depleted, accelerating the pathogenesis of CVD [48]. Furthermore, in other conditions that enhance oxidative stress, such as diabetes, dyslipidemia, hypertension, and aging, eNOS cofactors and substrates tetrahydrobiopterin (BH_4_) and L-arginine become depleted, leading to a phenomenon known as eNOS uncoupling [49]. In eNOS uncoupling, eNOS produces O_2_− instead of NO, which then reacts with NO (“scavenges it”) to create the harmful peroxynitrite radical (ONOO−) that oxidizes its scavenger reduced glutathione (GSH), a molecule that plays a crucial role against reactive oxygen species (ROS). The attenuated function of GSH and reduced NO bioavailability in atherosclerotic patients helps promote endothelial dysfunction and subsequently propagates the formation of atherosclerotic plaques.

Asymmetric dimethylarginine (ADMA), an endogenous eNOS inhibitor, may play an important role in the pathogenesis of atherosclerosis. ADMA is elevated in pathological conditions such as hypercholesterolemia and prolonged ischemia reperfusion [50]. By inhibiting eNOS and activating the angiotensin II-NADPH oxidase pathway of the renin angiotensin system, ADMA contributes to superoxide production and oxidative stress. A study by Willeit et al. found that patients with elevated levels of ADMA may be at an increased risk for developing CVD, coronary heart disease, and stroke [51]. It is therefore not surprising that ADMA has been associated with endothelial dysfunction among multiple organ systems, cardiovascular and noncardiovascular, as its presence is associated with increased levels of inflammatory cytokines, including IL-1β, TNF-α, IL-6, IL-10, IL-4, IL-2, and more [52]. Additionally, ADMA has been linked to the cell adhesion molecule VCAM-1, which aids in leukocyte and monocyte adhesion, and VEGF-A, a key mediator in the progression of atherosclerosis.

### 2.4. The Role of Connexins in Atherosclerosis

Connexins are proteins involved in cell-to-cell communication that form gap junctions and transmembrane channels known as hemichannels. There are five connexins expressed in ECs: Cx32, Cx37, Cx40, Cx43, and Cx45 [53]. Studies have shown that inflammatory stimuli in atherosclerosis affect the expression of these connexins, which play an active role in EC barrier integrity and function. Cx37, which is known to suppress VSMC proliferation, monocyte recruitment, and inhibit NF-κB, becomes downregulated by oxLDLs and factors such as TNF-α [54]. Consequently, its dysfunction enhances the development of atherosclerosis via monocyte adhesion to the ECs. Cx32 is equally important, reducing tissue factor (TF) expression to delay the coagulation process in atherosclerotic plaques. Similar to Cx37 and Cx32, the presence of Cx40 whose function is implicated in arterial vasodilation, becomes decreased by TNF-α [55]. On the other hand, Cx43 expression is upregulated in the early stages of atherosclerosis and downregulated in its late stages, with studies indicating a role in reducing plaque formation and neutrophil recruitment [56].

### 2.5. The Role of Inflammation

There are multiple inflammatory signaling pathways that have been implicated in the development and progression of atherosclerosis. In their review article, Kong et al. highlighted the additional presence of the proprotein convertase subtilisin/kexin type 9 (PCSK9), Notch, and Wnt signaling pathways [57]. The liver-produced PCSK9 downregulates low-density lipoprotein cholesterol (LDL-C) receptors (LDLRs) on the surface of hepatocytes, leading to increased LDL-C levels [58]. Induced inflammation in atherosclerosis activates the ROS/NF-κB/LOX-1/oxLDL axis, upregulating the expression of PCSK9, which further upregulates LOX-1 to induce the transformation of macrophages and VSMCs into foam cells, and CD36 to induce platelet activation and thrombosis.

The Wnt glycoproteins (19 in total) are involved in three different Wnt signaling pathways: the canonical (β-catenin-dependent) Wnt pathway, the noncanonical planar cell polarity (Wnt/PCP) pathway, and the Wnt-calcium (Wnt/Ca^2+^) pathway, all of which become activated when Wnt proteins bind to receptors of the Frizzled family (Fzd), sending a signal to the Disheveled protein (Dvl) in the cytoplasm [59]. Activation of the Wnt signaling pathways through Wnt1 and Wnt5a contributes to endothelial dysfunction and inflammation through the release of pro-inflammatory cytokines and the proliferation of ECs, while Wnt1 and Wnt4 have been shown to be involved in VSMC proliferation.

**Figure 3:**
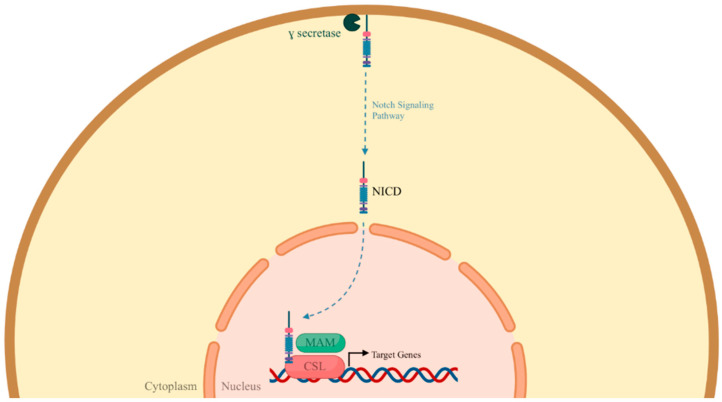
Intracellular portion of the Notch pathway. Once the intracellular domain of the Notch receptor (NICD) has been cleaved by the enzyme γ-secretase, it translocates to the nucleus to bind to transcription factors such as Mastermind-like proteins (MAMLs) and CSL for the activation of target transcription genes [62].

Finally, the Notch pathway has been implicated in atherosclerosis. There are four isoforms of Notch receptors (Notch1–4) in mammals that interact with ligands such as Jagged (JAG) 1 and 2 and Dll (Delta-like) 1, 3, and 4 [60]. The binding of either ligand to a Notch receptor in ECs will generate the cleavage of the extracellular domain of the Notch receptor by an A disintegrin and metalloprotease (ADAM), and the cleavage of its intracellular domain by a presenilin-dependent γ-secretase complex, resulting in its active form, the NICD (Notch intracellular domain). The NICD translocates to the nucleus and binds to, among others, the recombination signal binding protein-J (RBP-Jκ) and other factors, such as p300, to activate transcription genes (Figure 3) [57]. Disturbed flow in atherosclerotic lesions is sufficient to activate the JAG1-Notch4 pathway, as demonstrated in the study by Souilhol et al., which promotes atherosclerosis [61]. Notch1 signaling expression is also modulated by shear stress and is associated with leukocyte recruitment by promoting translocation of NF-κB [60].

### 2.6. The Role of microRNA and siRNA Therapies

MicroRNAs (miRNAs) are a class of small, noncoding ribonucleic acid (RNA) molecules that help regulate gene transcription and protein translation. There are thousands of unique microRNAs in the human genome. In the midst of multiple aforementioned negative factors, microRNAs aim to serve as important regulators in the progress of atherosclerotic plaques. For instance, miR-520c-3p helps regulate EC proliferation, apoptosis, and adhesion, while miR-181a-5p, miR-181a-3p, and miR-250b inhibit NF-κB activation [63]. miR-126 has both beneficial and disadvantageous effects. On the one hand, it can inhibit the apoptosis of vascular ECs by regulating the phosphatidylinositol 3-kinase/protein kinase B (PI3K/AKT) pathway and promoting EC angiogenesis, a process related to plaque growth and rupture (which is opposed by miR-92a). On the other hand, it aids in minimizing the inflammatory profile of atherosclerosis by reducing the macrophage and apoptotic cell content of plaques [64]. Like mir-126, mir-155 serves as a trigger for atherosclerosis, as its activation through inflammatory cytokines such as IFN-β and IFN-γ via TNF-α upregulates macrophage stimulation [65]. Therefore, the use of miRNAs, either in the form of anti-miRNA therapy to decrease the levels of miRNAs with pro-atherosclerotic properties or in the form of synthetic miRNAs to increase the levels of miRNAs with anti-atherosclerotic properties, may provide an opportunity in the future for regulating the progression of vascular disease [66].

Similar to the use of miRNAs as a potential therapeutic approach to atherosclerosis, the use of small interfering RNAs (siRNAs) has been recently proposed. siRNAs are double-stranded RNA molecules that bind and degrade target messenger RNA (mRNA) molecules [67]. One type of siRNA molecule that has been of particular interest is N-acetyl galactosamine (GalNAc)-conjugated siRNA. GalNac-conjugated siRNAs have been found to bind to a variety of target molecules, including PSCK9, angiopoietin-like 3 (ANGPTL3) and lipoprotein (a) [68]. ANGPTL3 is a protein that inhibits lipoprotein and endothelial lipases, resulting in elevated triglycerides [69]. Lipoprotein (a) also plays a role in cardiovascular disease by participating in arterial wall cholesterol deposition [70]. The incorporation of GalNac-conjugated siRNAs could thus aid in the inhibition of the early stages of atherosclerosis by reducing cholesterol levels and halting inflammatory stimuli that could propagate plaque development and instability.

### 2.7. The Role of VEGFs

Vascular endothelial growth factors (VEGFs) are growth factors that exert strong effects on endothelial cell proliferation, differentiation, migration, and growth. They are categorized into seven groupings: A, B, C, D, E, F, and placental growth factor (PlGF). PlGF and VEGF A, B, C, and D exist in humans and exert their effects through binding to endothelial growth factor receptors (VEGFRs), a type of tyrosine kinase receptor [71]. Two of these receptors, VEGFR1 and VEGFR2, are expressed on vascular endothelial cells and share proangiogenic functions, with VEGFR2 associated with increased vascular permeability and pro-inflammatory effects [72].

The VEGF family is upregulated in hypoxic conditions that increase the expression of transcription factor hypoxia-inducible factor-1 subunit alpha (HIF-1α), the latter of which promotes responses that increase oxygen levels and facilitate metabolic adaptation to hypoxia [73]. However, HIF-1α can also be increased in an oxygen-dependent manner. For example, the presence of ROS, cytokines, and lipopolysaccharides (LPSs) can act through pathways involving protein kinase C (PKC), IKK, and phosphoinositide 3-kinase (PI3K) to activate transcription factors NF-κB, specific protein 1 (SP1), and NF-E2-related factor 2 (NRF2) to regulate the expression of the HIF-1A gene. The expression of the HIF-1A gene is also regulated by the signal transducer and activator of transcription 3 (STAT3), which, through an auto-feedback loop, increases VEGF secretion and further activates STAT3 [74].

**Figure 4:**
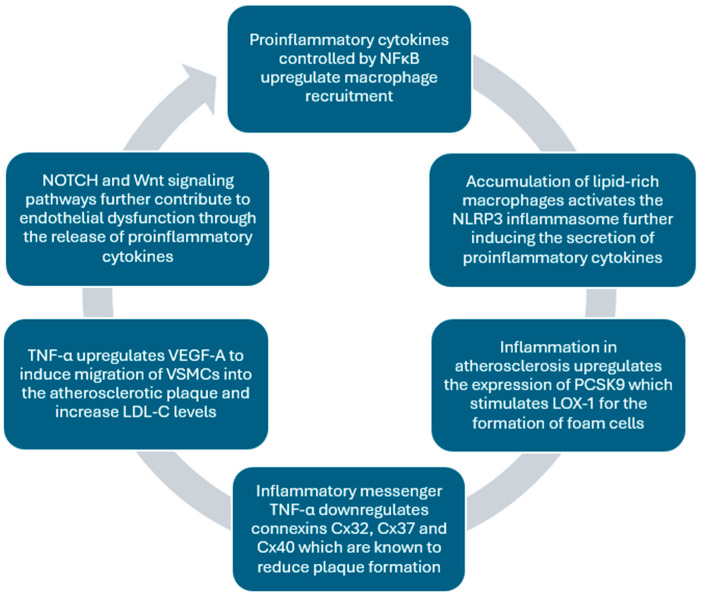
Summary of the inflammatory feedback mechanism in the initiation of atherosclerosis. Atherosclerosis is not just a collection of individual events but a positive feedback loop in which each step upregulates the previous one. The infiltration of LDLs into the tunica intima and the creation of lipid-rich macrophages in conjunction with inflammatory cytokines stimulate the progression of atherosclerosis.

The TNF-α-NF-κB-HIF-VEGF signaling cascade has exhibited clinical significance, and VEGF-A has been thoroughly studied for its strong connection to cardiovascular disease. It has been shown to stimulate plasma lipids by inhibiting the activity of lipoprotein lipase and increasing the triglyceride concentration in very low-density lipoprotein (VLDL) particles [75]. VEGF-A has also been associated with an increase in LDL-C. Additionally, VEGF-A, triggered by hypoxia and inflammation, upregulates the migration of VSMCs into the atherosclerotic plaque and the secretion of chemotactic substances for monocyte recruitment. The inflammatory feedback mechanism in atherosclerosis and the role of VEGF-A are highlighted in Figure 4 below.

VEGF-B binds to VEGFR1 expressed in vascular cells. VEGF-B, through its two main isoforms, VEGF-B167 and VEGF-B186, has a strong cardioprotective effect [76]. In addition to upregulating antioxidant genes such as Gpx1 (Glutathione peroxidase 1), it also upregulates angiogenesis and arteriogenesis after myocardial infarctions (MIs) through VSMC proliferation and EC migration. At the same time, it impairs the recycling of low-density lipoprotein receptors (LDLRs) to the plasma membrane, leading to reduced cholesterol uptake [77]. Reduced cholesterol uptake can lead to decreased levels of glucose uptake through negative feedback on the glucose transporter 1 (GLUT1), potentially exacerbating insulin resistance in diabetic patients. Like VEGF-B, PlGF exerts a cardioprotective effect. It helps improve the remodeling of the heart after an MI by increasing the vascular perfusion area and promoting angiogenesis at the site of the infarct [78].

VEGF-C binds to VEGFR-3 to induce lymphangiogenesis but can also exert some angiogenic effect through its binding to VEGFR-2 [79]. Glinton et al. additionally found that macrophages deficient in VEGF-C had elevated levels of pro-inflammatory marker, TNF-α, and reduced levels of arginase 1 (Arg1), which is associated with anti-inflammatory properties [80]. VEGF-D exerts a similar effect to VEGF-C through its binding to VEGFR-3, and blockage of this receptor is associated with retention of triglycerides and increased excretion of both triglycerides and free fatty acids (FFAs) [79].

## 3. Risk Factors

When looking at the components involved in initiating atherosclerosis, the influence of specific risk factors cannot be ignored. Epidemiological studies have found that although women are less likely to experience cardiovascular disease and myocardial infarctions compared to men, these differences become minimal when women are closer to the age of 70, and the risk for women surpasses men by the ninth decade of their lives [81]. One possible explanation for this change is that pro-inflammatory biomarkers like IL-1, IL-6, IL-18, TNF, and C-reactive protein (CRP) are increased in menopause and older patients. In one review article, Man et al. provided additional information to explain the general discrepancy between the sexes, showing that men have higher LDL and lower HDL levels compared to women among all age groups and that they comprise 80% of world smokers [82]. As a result, there is a greater incidence of thin-cap fibro-atheromas in men compared to women when both groups are under 75 years of age, around which time women start surpassing men. They also stated that the carotid artery intima-media thickness (IMT) was greater in men compared to women under 75 years of age and that men under 55 were more likely to have hypertension compared to women. Smoking-induced atherosclerosis is associated with not only ROS formation and reduced NO availability but also with activation of matrix metalloproteinases (MMPs) to promote plaque formation, pro-inflammatory cytokines, and the NLRP3 inflammasome, contributing to further endothelial dysfunction [83].

The link between hypertension and atherosclerosis is well established, with hemodynamic shearing forces intensifying during hypertension and inducing plaque rupture [84]. Thus, the adequate control of blood pressure is important in minimizing the risk of the development and progression of cardiovascular disease. In a European study on carotid atherosclerosis, Mancusi et al. found that participants with carotid plaques had higher systolic and lower diastolic blood pressures at baseline. The presence of carotid plaques was also significantly associated with an increased probability of uncontrolled blood pressure during follow-up after the effects of age, male sex, and presence of diabetes were controlled [85]. U.S. studies have also found that when looking at race, Hispanic, non-Hispanic African American, and Asian American individuals had lower blood pressure control rates compared to non-Hispanic Caucasian Americans [86]. Therefore, social determinants that affect these groups, such as access to healthcare, dietary habits, and socioeconomic status, can significantly influence the presence of hypertension and progression to atherosclerosis.

Obesity goes hand in hand with atherosclerosis and inflammation, with adipokines such as leptin, resistin, retinol-binding protein 4 (RBP4), angiopoietin-like protein 2, and monocyte chemoattractant protein-1, along with cytokines IL-6, IL-1β, IL-18, and TNF, activating macrophage recruitment and endothelial dysfunction in obese patients [83]. Moreover, atherosclerosis can be induced by diabetes mellitus, where glycosylated proteins and lipids known as advanced glycation end-products (AGEs) can be formed in the presence of high blood sugar levels [87]. These compounds can contribute to the progression of vascular disease in diabetes, given that they are metabolized slowly. Inflammatory cytokines in diabetic patients can further induce the formation of specific miRNAs, namely miR-146 and miR-126, to propagate inflammatory pathways. In diabetic patients with chronic kidney disease (CKD), LDL levels are commonly elevated, and so is oxidative stress [88]. Oxidative stress in CKD patients can cause accelerated senescence and death in endothelial and endothelial progenitor cells, as well as contribute to enhanced atherogenesis, which can further accelerate CKD progression [89].

Lack of regular exercise is another predisposing factor to atherosclerosis. Indeed, regular exercise has been associated with increased HDL levels and reduced insulin resistance and LDL levels (when combined with weight loss) [90]. However, studies show that vigorous physical activity increases the risk of sudden cardiac death and myocardial infarction in patients with known heart disease. Similarly, studies have found that exercise intensity, not volume, is associated with the progression of coronary atherosclerosis in middle-aged and older athletes (50 to 60 years of age) [91]. One suggestion for this discrepancy is that high-intensity exercise increases catecholamine levels that trigger a rise in blood pressure, arterial mechanical stress, and pro-inflammatory changes in monocytes. Hence, individuals interested in reducing their risk of developing or worsening cardiovascular disease by wishing to incorporate exercise would benefit from individualized exercise plans.

Emerging and less recognized risk factors contributing to atherosclerosis and cardiovascular manifestations include genetic predispositions, gut microbiota and inflammation. A review by Björkegren et al. indicated that over 200 chromosomal loci have been identified for CAD and highlighted a Y chromosome haplotype containing the UTY gene that may increase cardiovascular risk [92]. In addition to genetics, the authors furthermore drew attention to gut microbiota, pointing that individuals who consume diets rich in choline or carnitine, and patients with kidney disease, have elevated levels of trimethylamine N-oxide (TMAO), an organic compound that has been linked to increased platelet reactivity and vascular inflammation, thereby accelerating the development and progression of atherosclerotic disease.

Chronic inflammation is a critical driver of CVD, particularly in patients with autoimmune conditions such as rheumatoid arthritis (RA), systemic lupus erythematosus, and psoriatic arthritis. Chronic inflammation in RA has been found to predispose patients to not only an increased risk for CVD compared to the general population due to the presence of exacerbated arterial stiffness and increased systemic atherosclerosis, but also to a greater risk of myocardial infarction [93]. Although additional risk factors such as hypertension, smoking, dyslipidemia and sedentary lifestyle may predispose patients with autoimmune conditions to the development CVD, immune dysregulation in conjunction with an increased inflammatory burden through the presence of pro-inflammatory cytokines like Il-6 and TNF-α, accelerates endothelial damage and plaque rupture [94]. For this reason, management and reduction of CVD in these populations entail both lifestyle modifications and anti-inflammatory therapies like disease modifying antirheumatic drugs (DMARDs), which include both TNF-α inhibitors and methotrexate, to reduce atherogenesis and cardiovascular events. Furthermore, research focusing on adaptive immune cells, such as CD4+ and CD8+ T-cells and pro-atherogenic B-cell subsets (particularly the B-2 subset), offers promising therapeutic avenues for reducing inflammation and plaque instability [95].

Nonetheless, clinicians aiming to reduce CVD development and progression should be aware of the possibility of residual CVD risk, meaning the risk of patients developing persistent CVD events despite receiving treatment and targeting risk factors such as LDL cholesterol, blood pressure, and glycemia [96]. Reijnders et al. identified additional molecular factors contributing to residual risk, including ANGPTL3, lipoprotein (a), and lipoprotein-bound phospholipase A2 (Lp-PLA2), which is an enzyme family responsible for the hydrolysis of oxidized phospholipids on LDL particles [97]. Additional identified molecular factors with important implications are apolipoprotein B (apoB), which is present in all atherogenic lipoproteins such as lipoprotein (a), LDLs, VLDLs, and chylomicrons, and apolipoprotein C-III (apoC-III), an inhibitor of lipoprotein and hepatic lipases. They listed fibrates, which decrease triglycerides and reduce available fatty acids for VLDL synthesis and secretion, mipomersen, an inhibitor of apoB formation, darapladib, an Lp-PLA2 inhibitor, GalNAc-conjugated volanesorsen, an apoC-III antagonist, and the concomitant use of clopidogrel and rivaroxaban as potential promising therapies for reducing CVD risk and preventing thrombotic events. However, they also stated that additional risk factors such as advanced age, variability of lipoprotein (a) among ethnic groups, and indication for dialysis among CKD patients, may be further contributing to the inability to lower CVD risk. Additionally, inflammation in the form of elevated high-sensitivity C-reactive protein (hs-CRP) and cytokines IL-1β and IL-6, is considered an important nonlipid residual risk factor [98]. Hence, clinicians will be tasked to consider a multifactorial contribution to CVD risk outside of the standard risk factors and treatment regimens.

## 4. Chronic Coronary Disease (CCD) Guidelines

As the name suggests, CCD, a direct byproduct of atherosclerosis, is a chronic disease, and therefore, it is imperative to be informed of the up-to-date guidelines. In 2023, Visani et al. published the American Heart Association (AHA) guidelines regarding the management of patients with CCD [99]. The guidelines include weight management techniques, lipid-lowering strategies, blood pressure control, and interventions for specific populations such as women and individuals with valvular problems or CKD. The AHA “compiled” these guidelines into ten take-home messages [100]. These are summarized in Table 1.

The AHA guidelines can be compared to or used in parallel with those from the European Society of Cardiology (ESC) when considering approaches to the management of CCD patients. Like the guidelines by the AHA, the ESC guidelines emphasize specific key points. These are detailed in Table 2 [101].

## 5. Conclusions

Multiple factors on a molecular and social level can predispose patients to the initiation, development, and progression of atherosclerosis. A pro-inflammatory environment created through multiple positive feedback loops accelerates the development of atherosclerotic plaques. Although the development of atherosclerosis has been extensively studied over the years, new emerging studies have shed light on the role of additional factors, including that of connexins and the VEGF family. The role of atherosclerosis in the development of CAD and CVD, has further established CAD as a chronic disease that necessitates the transition to a new medical terminology that encompasses its long-term importance and effects (that of chronic coronary disease), and the establishment of guidelines that will continue to be updated based on novel research.

## Figures and Tables

**Table 1:** The ten take-home messages from the 2023 American Heart Association guidelines regarding the management of patients with CCD.

Ten Take-Home Messages from the 2023 American Heart Association Guidelines Regarding the Management of CCD
Team-based, patient-centered care
Incorporation of nonpharmacologic therapies such as diet and exercise
Use of habitual physical activity for CCD and cardiac rehabilitation for eligible patients
Administration of sodium–glucose cotransporter two inhibitors and glucagon-like peptide-1 receptor agonists in CCD patients with or without diabetes
Use of either a calcium channel blocker or a beta-blocker as first-line therapy for angina while avoiding the use of beta-blockers in CCD patients in the absence of myocardial infarction in the past year and left ventricular ejection fraction ≤50% or another primary indication for beta-blocker therapy
Use of statins as first-line therapy for lowering lipids in CCD patients
Administration of dual antiplatelet therapy for a shorter duration is safe and effective when the risk of bleeding is high and the ischemic risk is low to moderate
The utilization of supplements such as fish oil, vitamins, and omega-3 fatty acids is not recommended due to the lack of benefit in reducing cardiovascular events
Routine periodic anatomic or ischemic testing without a change in clinical or functional status is not recommended to guide decision-making in CCD patients
The usage of e-cigarettes is not recommended as first-line therapy for smoking cessation due to a lack of long-term data regarding safety and risks

**Table 2:** Current 2024 European Society of Cardiology Guidelines for the Management of Chronic Coronary Syndromes.

European Society of Cardiology Guidelines for the Management of Chronic Coronary Syndromes
The term chronic coronary syndrome (CCS) is used to describe clinical presentations of coronary artery disease (CAD) during stable periods, before or after acute coronary syndrome (ACS) events
Implementing a general clinical evaluation, cardiac examination, and diagnostic testing should be emphasized to establish the diagnosis of CCS along with the use of lifestyle modifications and disease-modifying medications
The employment of non-invasive anatomic or functional imaging as first-line diagnostic testing of suspected CCS
Coronary computed tomography angiography (CCTA) should be preferred as an initial test to rule out obstructive CAD. The use of invasive coronary angiography (ICA) can be considered in patients with a very high clinical likelihood of obstructive CAD, symptoms unresponsive to medical therapy, angina at a low level of exercise, and/or high-event risk
The use of a single antiplatelet like aspirin or clopidogrel is recommended as part of the long-term therapy for CCS patients with obstructive atherosclerotic CAD
CCS patients with functionally significant multivessel CAD or reduced left ventricular function and ischemic cardiomyopathy would benefit from myocardial and surgical revascularization, respectively, over guideline-directed medical therapy (GDMT) alone when taking into consideration overall survival and reduced mortality
